# A task-specific architecture with multi-scale attention and shape-aware loss for strawberry phenophase recognition in complex fields

**DOI:** 10.3389/fpls.2026.1888504

**Published:** 2026-06-22

**Authors:** Shilin Li, Shangjian Guo, Nan Yang, Lili Sun, Shujuan Zhang, Fuzhong Li

**Affiliations:** 1 Faculty of Software Technologies, Shanxi Agricultural University, Jinzhong, China; 2 College of Agricultural Engineering, Shanxi Agricultural University, Jinzhong, China

**Keywords:** agricultural robotics, object detection, phenological period detection, precision agriculture, selective picking

## Abstract

To address the challenges of recognizing small strawberry targets and achieving accurate phenological perception in complex field environments, this paper proposes a novel end-to-end lightweight detection architecture named HCMS-Net. The backbone is a Residual Efficient Layer Aggregation Network (R-ELAN) enhanced with a Multi-Scale Convolutional Attention (MSCA) mechanism, which emphasizes subtle color and texture variations to differentiate key phenological phases. For feature fusion, hypergraph convolution (from HyperC2Net) and a Mixed Aggregation Network (MANet) are incorporated, modeling the clustered morphology of strawberries and strengthening the representation of sparse small fruits. The detection head incorporates a lightweight Conv2Former module to capture long-range dependencies and spatial contextual information across growth stages, thereby enhancing the model’s capacity to represent continuous phenological changes. A Shape-Normalized Wasserstein Distance (Shape-NWD) loss is introduced to stabilize optimization against minor pixel deviations. Experimental results demonstrated that HCMS-Net achieved a mean average precision (mAP) of 94.9% and an F1-score of 90.0%. Specifically, the average precision (AP) values for the flowering, young fruit, green fruit, veraison, and mature fruit stages reached 99.3%, 88.3%, 90.9%, 97.0%, and 98.2%, respectively. Heatmaps confirmed HCMS-Net’s precise attention focus across all five phenological stages, effectively suppressing irrelevant backgrounds. Compared to ten mainstream detectors, HCMS-Net surpassed alternatives such as RT-DETR and the YOLOv5n to v13n by 3.4–8.0 percentage points in mAP. It even surpassed YOLOv12s by 2.7 percentage points, while containing only 32.86% of its parameters. The model offers high accuracy and efficiency for phenological period detection, supporting selective harvesting and intelligent agricultural management.

## 1 Introduction

Strawberry is widely grown all over the world and have high nutritional and economic value. Strawberry has its corresponding phenological stage in different stages of growth, which requires targeted management measures, such as scientific fertilization, precision irrigation, bud cutting and pruning, disease control, batch picking and so on, to effectively reduce planting and picking costs and improve production efficiency. In this study, referring to the Standardized Technical Regulations for Strawberry Production (NY/T 2693-2015) issued by the Ministry of Agriculture and Rural Affairs and the Guideline for Strawberry Production Technology (GB/Z 26575-2011), the different growth stages of strawberries were divided into five phenological stages: flowering, young fruit, green fruit, color turning, and ripening stages (de Oca et al., 2025; An et al., 2024). With the increasing market demand, strawberry planting scale has also expanded. Whether it is planted in traditional greenhouses or on three-dimensional elevated shelves, it is necessary to carry out corresponding management according to different phenological stages of strawberries to achieve accurate detection and batch picking. In addition, the fruit form of strawberry is small, and it is easy to have the problem of feature loss or even drowning in object detection. There is a lack of enough fine-grained features for the model to learn, which increases the difficulty of object detection. At the same time, some phenological fruits are easy to be confused, and it is often very difficult to determine the phenological stage of strawberry, the results are not accurate enough and the workload is large. With the advancement of computer vision technology and smart agriculture, strawberry phenological stage recognition has become the primary content of automated picking. Therefore, phenological stage identification technology can change the traditional planting mode that farmers rely on personal experience, and timely perform agricultural operations such as watering, fertilization, fruit thinning, pest control and harvesting according to more accurate phenological stages, which is the core driving force for reducing costs and increasing efficiency, improving quality and increasing income (Ren et al., 2025).

The continuous advancement of AI has enabled deep learning methods to be widely applied in fruit detection tasks (Huang et al., 2026; Chen et al., 2025; Wu et al., 2026), which has provided great help for the realization of strawberry phenological stage detection. However, strawberry phenology detection still faces multiple challenges. In addition to being limited by the complex and unstructured agricultural environment, small targets are prone to information loss, which makes detection difficult. In this context, improving the detection ability of the model for small targets has become the key to achieve accurate identification of strawberry phenology.

Small object detection (SOD) often leads to weak features or even missing features, and the mismatch between receptive field and target size due to less pixel information, low image resolution, occlusion and overlap. Aiming at the problem of feature loss easily existing in the process of SOD, Hu et al. (2025) proposed the FSEM mechanism and IKLD loss function to compensate for the informational deficit, which increased the detection accuracy by 2.2 percentage points. However, this method does not effectively use the context information, resulting in ignoring the structural features of the image. Therefore, Tong and Wu (2024) proposed a single-layer DFLM and FFB to activate the multi-scale receptive field to better integrate the deep features and shallow features. However, this model will blur the reliability of context information when dealing with small target noise images, so Li et al. (2025a) used AConv to replace DWConv in VSS module to effectively suppress noise, and the final AUC value reached 0.9997. On the other hand, aiming at the insufficient alignment of fine-grained features, Yu et al. (2025) designed the ADRConv structure, which utilizes second-order central difference to extract small object features for representing meaningful context, resulting in a 4.3% increase in detection accuracy. Similarly, MAFNet (Wang et al., 2025a) aligns features of different scales based on SAFB and MSB modules, which enhances the recognition ability of small targets in low-resolution images, and the final detection accuracy is 93.94%. Also targeting infrared of SOD, Lu et al. (2025) proposed the nested feature pyramid structure, and improving suppression of distracting objects. However, the model’s high complexity and large parameters, which hinders lightweight deployment in practical applications. To this end, many researchers have studied the lightweight of models. For example, Jing et al. (2024) adopted deformable convolutions to accurately localize boundaries of objects, which reduced the computational cost and increased the mAP by 0.8%. On the basis of optimizing the convolution operation, Yang et al. (2025) integrated an information fusion strategy, which emphasizes critical information and suppresses redundancy, thereby improving computational efficiency. Some scholars focus on lightweight backbone network. Zhang et al. (2025) used MobileNetV3 to replace the original backbone network to reduce model parameters, and the final model size was 3.9MB, which was 72.9% lower than the original network. In addition to network structure optimization, technologies such as structured pruning and knowledge distillation also develop rapidly. For example, Ruan et al. (2025) used knowledge distillation technology based on Segment Anything model and replaced high-parameter modules with lightweight image modules, and the final model volume was compressed by 42.7%. On this basis, more scholars combined knowledge distillation and model pruning technology, such as Zhang et al. (2025) achieved 94.3% parameter reduction through this fusion method, and integrated the multi-scale fusion network, and the accuracy of safflower picking point identification reached 96.7%.

On the other hand, researchers have achieved remarkable results in the identification of phenology. Liu et al. (2024) used the average pooling layer to replace the original maximum pooling layer based on ResNet, and used the residual connection mechanism to identify different phenological stages of wheat, and the overall recognition accuracy reached 94.4%. Similarly, Zhou et al. (2025) integrated 3D height features with 2D image features based on VanillaNet to classify wheat phenological stages into five categories, including tiller, jointing & heading, heading, flowering & filling, and maturity stage. The final accuracy of each phenological stage was 0.96, 0.88, 0.66, 0.87 and 0.96, respectively. On this basis, Zhu et al. (2025) introduced self-attention and cross-attention mechanism into the feature enhancement layer for apple detection and maturity classification, and the experimental results show that the mAP reaches 72.8%. However, in the case of overlapping occlusion, multiple edge confusion is easy to cause irregular features. Therefore, Mohsan et al. (2025) used HardSwish nonlinear activation function to replace the attention layer in Transformer, which improved the accuracy of the model by a small margin without increasing the inference time. The recognition accuracy of unripe, ripe and overripe state of avocado reaches 96.6%, 98.3% and 93.3%, respectively. However, in high-density occlusion scenes, this model will cause feature norm decay, resulting in low variance representation, and even feature submersion. Therefore, Xie et al. (2025) introduced the MLCA attention mechanism, and adopted the Mask Generative Distillation (MGD), enhancing the capture of small-object features. Further improving feature extraction for very small targets, Wang et al. (2025b) proposed Multiple Branch Block Fusion module (DBFusion) and Multi-scale Multi-head Self-Attention (MMSA) based on RT-DETR network, strengthening local-global feature interaction, detecting five phenological stages of pomegranate with a mAP of 93.4%. Li et al. (2025b) maintained fine-grained features by rearranging channels based on MSBlock module, which was used to accurately identify the flowering stage of kiwi, and the result showed that mAP reached 94.07%. Inspired by this, Li et al. (2025c) introduced Asymptotic Feature Pyramid Network (AFPN) into the state-space model SSM to retain critical features and enhance the integration of early inflorescence details. The mAP of early inflorescences increased by 13.93 percentage points. However, this model only considers the identification of early grape growth stages, and the phenological stage identification of the whole grape growth cycle still needs to be improved. Therefore, Lyu et al. (2025) employed a conditional WGAN for data augmentation, and detected the complete phenological stage of fruits, achieving 91% accuracy. Collectively, these studies demonstrate notable advances in deep learning for agricultural phenological detection, providing an important reference for our subsequent detection of strawberry phenology.

Based on the self-built “Hongyan” strawberry data set of different phenological stages, this study proposes a HCMS-Net model for the detection of strawberry phenological stages, aiming at the two detection difficulties of SOD and phenological stage identification, and makes the following improvements (see **Table 1** for details):

**Table 1:** Mapping from task challenges to HCMS-Net design components.

Task challenge	Visual manifestation	Required functional property	Module
Fine-grained inter-class similarity	Subtle calyx, color differences	Multi-scale fine-detail extraction	HyperC2Net
Occlusion & complex background	Leaves cover >50% of fruit	Local fragment attention + global context	MSCA + Conv2Former
Tiny target localization instability	Budding stage<20×20 px	Scale-stable gradient & shape metric	Shape-NWD Loss

1. In this study, the R-ELAN was used as the backbone and integrated into a multi-scale convolutional attention MSCA, which imposes differential weights on features at different spatial locations to realize the selection and enhancement of spatial adaptive features.
2. The hypergraph convolution of HyperC2Net was integrated in the neck to establish the association between small target features and their contexts, and a hyperedge structure covering small target groups was constructed to use the common features to make up for the limitations of single target features.
3. The detection head part integrated the Conv2Formers module to replace the self-attention mechanism by convolutional modulation operation, which establishes long-range dependencies across regions and more coherently captures the spatial context information between targets at different growth stages, thus enhancing the model’s ability to characterize continuous phenological changes.
4. The Shape-NWD loss function was introduced to alleviate the optimization instability problem caused by small pixel deviation in SOD through the shape-aware normalized distance measure, and this improved the model’s adaptability to scale and morphological variations.

## 2 Materials and methods

### 2.1 Dataset acquisition

**Figure 1:**
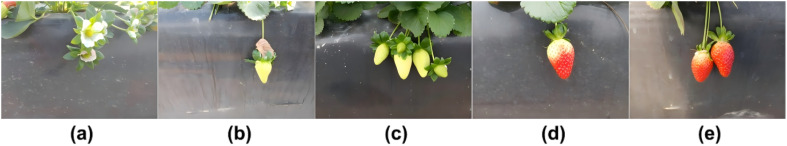
Strawberry phenological period dataset. **(a)** Flower **(b)** Early **(c)** Green **(d)** Turning **(e)** Ripe.

The image data were obtained from the “Hongyan” strawberry of Juxin Modern Agricultural Park in Taigu District, Jinzhong City, Shanxi Province, and the acquisition equipment was a Japanese Nikon D7500 camera and a vivo iQOO Neo6 SE smartphone. Images were taken over the entire growth cycle, from March 2025 to May 2025, at three time periods (7:30-09:00, 11:00-13:00, 17:00-18: 30) multi-conditions (such as illumination, occlusion, overlap, etc.) and multi-angle shooting, a total of 5670 images with resolutions of 4608×3456 and 3840×2160 are collected as the data set. **Figure 1** illustrates representative examples of five phenological phases.

To ensure the completeness of phenological stages and the diversity of the dataset, data were collected in the orchard setting on alternate days under varying conditions of illumination and occlusion. Manual annotation of images from each phenological stage was performed using the LabelImg tool, with detailed criteria (source: Standardized Production Technical Regulations for Strawberry.) provided in **Table 2**.

**Table 2:** Phenological period classification criteria.

Level	Maturity	Evaluation standards	Smell
1	Flower	The white petals and yellow stamens of the delicate flower cluster in the center of the plant.	A faint and sweet floral fragrance.
2	Early	The fruit is very small, dark green or bluish-green, with a surface densely covered by distinct white or light yellow velvety hairs.	The refreshing scent of the plant stems and leaves.
3	Green	The fruit volume increases rapidly, turning light green or yellowish-green, with the surface fuzz gradually decreasing or shedding.	It began to emit a pungent, sour, and astringent plant odor.
4	Turning	The sunlit side first fades from green to yellowish-white, then rapidly appears red or pink, covering less than three-quarters of the surface.	The sweet aroma of strawberries began to evaporate.
5	Ripe	The fruit is uniform, bright and glossy deep red and more than three quarters of the area.	The aroma of strawberry is rich and unique.

The annotation was conducted by a team of three trained annotators under the supervision of an agricultural expert with over five years of experience in strawberry phenotyping. Annotations were performed using the LabelImg tool, and each annotator independently labeled a non-overlapping subset of the images. Images were not annotated if the strawberry fruit was obscured by leaves covering more than two-thirds of its surface area, and the phenological stage could not be inferred with confidence from the visible portion. A fruit was labeled as the mature stage if the red surface area exceeded three-fourths; otherwise, it was assigned to the color-turning stage. The final dataset comprises five categories: Flower (flowering stage), Early (young fruit stage), Green (green fruit stage), Turning (color-turning stage), and Ripe (mature stage). The dataset was randomly split into training, validation, and test sets in an 8:1:1 ratio. The distribution details across these sets are provided in **Table 3**. Here, “Detection Grid Points” refer to the number of spatial locations on the feature map where the anchor-free head predicts bounding boxes.

**Table 3:** Distribution of data sets.

Data set	Image number	Detection grid points					
		Flower	Early	Green	Turning	Ripe	Total
Training	4592	4701	5589	3106	3570	4357	21323
Validation	511	518	643	376	413	472	2422
Test	567	559	773	373	441	534	2680
Total	5670	5778	7005	3855	4424	5363	26425

### 2.2 The structure of HCMS-Net

**Figure 2:**
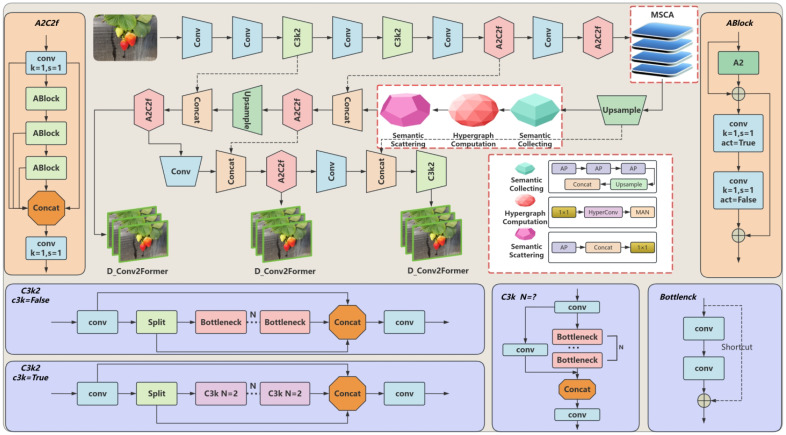
Network structure diagram of HCMS-Net. MAN, MANet network; AP, Average Pooling operation.

To enhance the detection accuracy of strawberry phenological stages, this study developed an HCMS-Net model based on R-ELAN and HyperC2Net networks, as illustrated in **Figure 2**.

Traditional attention mechanisms are often constrained by computational costs, which limits their applicability in real-time object detection. YOLOv12 also uses R-ELAN as the backbone network, and its A2 module reduces the attention complexity in a simple and efficient way. By dividing the feature map into multiple regions in the horizontal or vertical direction, the complex window division operation is avoided, so as to accelerate the attention calculation. The traditional global attention calculation is transformed into local attention calculation, which significantly reduces the computational complexity. Although the attention is calculated locally, the A2 module can still maintain a large receptive field because the feature map is divided into multiple regions, so as to capture the global context information. Therefore, A2 module only needs feature map division and local attention calculation to avoid complex window division and inversion operations. In order to extract features flexibly, the C3K2 module is used to grasp the key features in the image. The branch parallel structure is used to reduce redundant calculations, and the flexible convolution kernel is used to capture the target features. In the channel attention part of A2C2f module, the global information of each channel is obtained by global average pooling, and the dependencies between channels are learned through two convolutional layers. Finally, the channel weight map is generated. The spatial attention part captures local spatial information through the convolution operation. These two attention maps are multiplied together and then applied to the original features, achieving adaptive feature enhancement. Simultaneously, the cross-scale feature fusion part of A2C2f performs weighted integration enabling the model to attend to both local details and global context.

The specific details include: (1) In the backbone, after the final A2C2f block, we embed a MSCA layer at layer 9 to enhance multi-scale contextual attention. (2) The neck performs multi-scale pooling and upsampling to construct a fused feature map, which is then processed by a HyperC2Net hypergraph module (HyperComputeModule) followed by the MANet blocks for higher-order feature refinement. (3) In the detection head, a Conv2Former module is inserted before the final decoders to capture long-range dependencies and suppress background noise. (4) The regression branch is supervised by the Shape-NWD loss (α=0.5, β=0.5) instead of CIoU, providing scale-stable gradients for the tiny strawberry targets.

### 2.3 Hypergraph-based cross-level and cross-position representation network

In the field of computer vision, how to mine high-order semantic associations from low-level features to support semantic understanding has always been a key and challenging research direction. HyperC2Net (Feng et al., 2024) gets rid of the traditional grid structure and allows complex high-order interactions across levels and locations. In this study, it was integrated with the neck network of YOLOv12. HyperC2Net incorporates semantic features at three different scales {N3, N4, N5}, corresponding to small-scale, mesoscale, and large-scale detections. It utilizes hypergraph computation to capture complex high-order relationships by performing a channel cascade of five base features to synthesize cross-level visual features. A hypergraph G={V, E} is generally defined by its vertex set V and hyperedge set E. The construction of the hyperedge set can be formulated as E = {ball(v, e) | v∈V}, where ball(v, e) = {u | ||x_u_ - x_v_||d<e, u∈V} denotes the set of neighboring vertices for a given vertex v. Here, ||x_u_ - x_v_||d represents a distance function. In computation, the hypergraph G is commonly represented via its incidence matrix H, expressed as follows see Equation 1 for details:

(1) $$H_{ij}=h\left(v_{i}\;,e_{j}\right)=\{\begin{array}{c} 1,\;\;\;\;\;if\;v_{i}\in e_{j};\\ 0,\;\;otherwise. \end{array}\;\;$$

**Figure 3:**
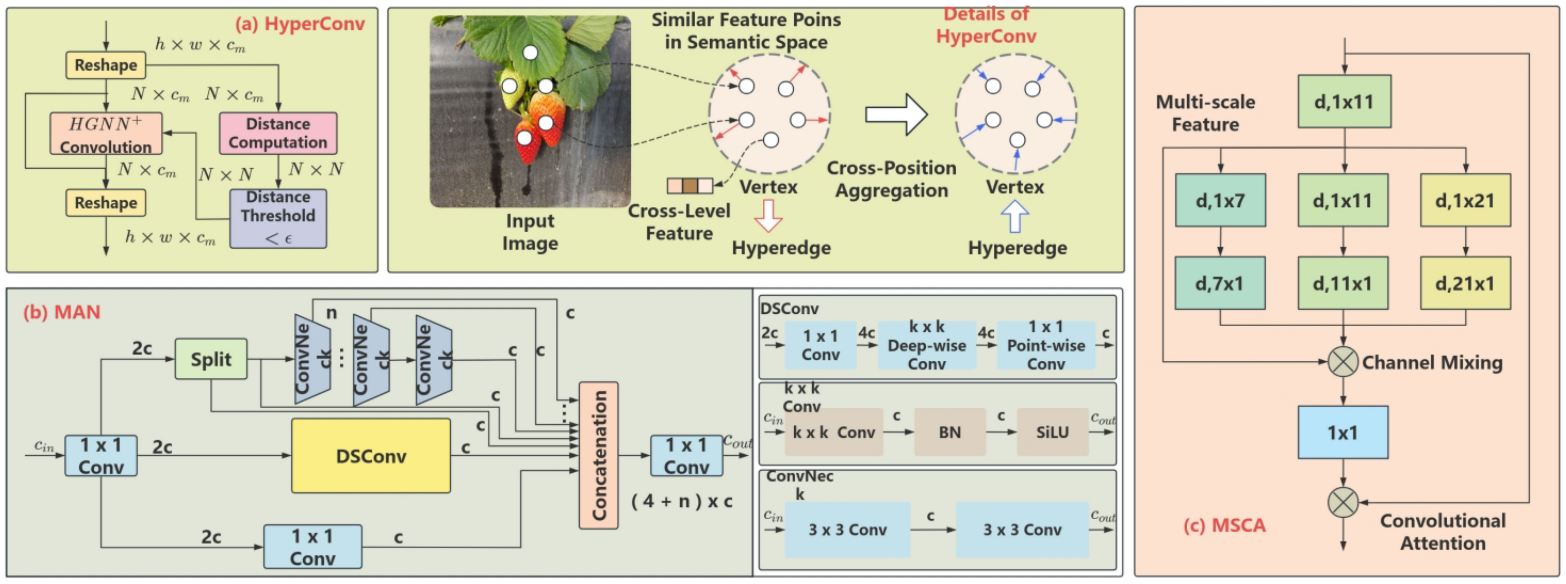
Structural diagrams of HyperC2Net and MSCA. **(a)** HyperMap convolutional structure diagram. **(b)** MANet structure diagram. d, k1×k2 denotes a deep convolution with kernel size k1×k2. **(c)** MSCA structure diagram.

In the traditional propagation mechanism of graph convolution, information is propagated from node to neighbor nodes and diffused hop by hop to further nodes. However, it will face the problem of information attenuation, which leads to the loss of details in deep features and the lack of semantics in shallow features. Therefore, HyperC2Net introduces hypergraph convolution to implement high-order message passing on hypergraph structures, which is a hypergraph-based graph neural network operation for handling higher-order relations and complex data structures. See **Figure 3** for the detailed structure.

Different from traditional graph convolution, hypergraph convolution connects multiple nodes through hyperedges, and information is directly synchronized among all nodes within the hyperedge, which can more flexibly model nonlinear high-order correlations (see **Figure 3a**). In the field of object detection, hypergraph convolution can realize the direct interaction of objects at any distance through a single-layer hyperedge connection, and has the ability to model long-range dependence, so as to realize the semantic association from local to global. At the same time, the hypergraph convolution can connect the small target with the relevant context, and construct the group hyperedge of the small target to compensate for the lack of individual features, so as to realize feature enhancement and collaborative detection. To further enhance feature extraction within the hypergraph computation process, the hybrid aggregation network MANet (see **Figure 3b**) is introduced in the hypergraph calculation process, which is an extension of YOLOv8 infrastructure. This method enriches the feature information flow and strengthens the semantic depth implied by the basic features.

### 2.4 Multi-scale Convolutional Attention module

Conventional convolutional neural networks often rely on fixed receptive fields, which lack the flexibility to capture contextual information across varying scales in an image, and the global attention mechanism often brings huge computational overhead. In order to balance representation ability and computational efficiency, and achieve more flexible feature fusion and selection, this paper introduces a MSCA module (Guo et al., 2022), as shown in **Figure 3c**.

The MSCA design combines local information aggregation, multi-scale context extraction and channel relationship modeling to enhance feature discrimination while maintaining low computational complexity. The MSCA consists of three components: a depthwise convolution for aggregating local information, multi-branch depthwise strip convolutions for capturing multi-scale context, and a 1×1 convolution for modeling inter-channel relationships. We added MSCA to the backbone and achieved remarkable results. The MSCA module employs convolutional kernels of varying sizes (e.g. 7×1, 11×1, 21×1, etc.) to perform multi-scale feature extraction. Following the multi-scale convolution operations, features from different scales are integrated via convolutions, thereby improving feature fusion. Finally, MSCA used convolution operation to capture the key regions in the image, and weighted the features of different positions to achieve spatially adaptive feature selection and enhancement. Moreover, this MSCA mechanism can automatically adjust the focus according to different tasks, so that a single model has multi-task adaptability.

### 2.5 A simple transformer-style ConvNet for visual recognition

The existing detection heads mainly rely on the traditional convolution operation for feature aggregation. Although convolution performs stable in local feature extraction, it is still weak in modeling long-distance dependence and global context information. Traditional feature fusion methods usually concatenate or add multi-level features element-by-element, which ignores the spatial consistency of different scales. Especially in complex scenes, when the target scale is variable and the background interference is strong, it is difficult to fully capture the complex relationship in space by simply relying on stacked convolutional layers. It will further lead to small targets being submerged by large-scale semantics and missed detection, and the edge of large targets is pulled away by small-scale noise, thus restricting the detection accuracy. We note that recent Vision Transformer-based methods have significantly enhanced global modeling capability through self-attention mechanisms. However, their computational complexity grows quadratically with image resolution, which becomes an obvious bottleneck in high-resolution detection tasks. Conv2Former (Hou et al., 2024) provides a compromise. It replaces traditional self-attention with convolutional modulation operations, which can implicitly establish long-term dependencies with large kernels (e.g. 11×11) while maintaining linear computational complexity.

For the improvement of the detection head part, this algorithm mainly integrates the Conv2Former module into the Detect module to perform feature fusion to obtain D_Conv2Former. Specifically, we use the convolutional modulation operation of Conv2Former to enhance the representation ability of features by Hadamard product of the spatial weights extracted by a deep convolution with the value features. Different from traditional self-attention ViTs, the core idea is to use convolution operation to build the network. This improvement has several obvious advantages: (1) When dealing with high-resolution images, this method is more suitable for high-resolution visual recognition design than the self-attention mechanism due to the use of large-size convolution kernels. When the image resolution increases, the computation does not grow quadratic with the Transformer model, but linearly. (2) Compared with the original Detect module, D_Conv2Former can more naturally integrate global context information, helping the detection head to better distinguish the foreground from the complex background. It is found that the recall rate of the detection head for occluded targets and small targets is significantly improved after integrating Conv2Former, which is probably because the receptive field expansion caused by the large convolution kernel allows the network to see further pixel relationships.

### 2.6 Shape-Normalized Wasserstein Distance

Existing bounding box regression methods (IoU, GIoU, and CIoU), primarily rely on the geometric relationships between predicted and ground-truth boxes, for instance, by calculating loss functions based on overlap area and center-point distance. However, these approaches often overlook the impact of the bounding box’s shape and scale on regression results. This issue becomes particularly pronounced in SOD tasks. Traditional loss functions fail to adequately accommodate this sensitivity, leading to reduced localization accuracy and insufficient detection stability. SOD is special. Because the target occupies very few pixels in the image, its features are often fuzzy and incomplete, which brings obvious difficulties to accurate recognition and location. In fact, the shape disparity and scale variation among regression samples can significantly affect the IoU value under the same position deviation: for small-scale bounding boxes, a slight deformation or offset may cause large fluctuations in IoU. This suggests that in the detection framework for small objects, the bounding box regression loss must take shape and scale into account, so as to introduce stronger geometric perception in the loss calculation and improve the discrimination of the model to subtle positioning errors. Therefore, we used a Shape-NDW (Zhang and Zhang, 2023) to enhance the spatial modeling ability of small objects.

In order to better deal with the influence of shape and scale factors on the regression results in bounding box regression, especially in SOD tasks, Shape-NWD integrates the concept of Shape-IoU into Normalized Wasserstein Distance (NWD). Specifically, Shape-IoU introduces a scale-related factor and shape-aware weighting coefficients—namely, the horizontal weight ww and vertical weight hh derived from the ground-truth box geometry—into the loss computation. NWD alone provides accurate center-of-mass guidance but produces weak shape correction, leading to elongated boxes. Shape-IoU alone corrects shape well but fails to provide sufficient positional gradients when the initial box is far from the target. Shape-NWD integrates both: in the early training phase, NWD dominates and rapidly pulls the box toward the target; in the later fine-tuning phase, Shape-IoU takes over to precisely refine the box boundaries.

Shape-NWD combines a shape-aware term (Shape-IoU) and the Normalized Wasserstein Distance (NWD). Shape-NWD is then defined as follows see Equations 2–4 for details:

(2) $$B=\frac{{\left(w-w_{gt}\right)}^{2}+{\left(h-h_{gt}\right)}^{2}}{weigh^{2}}$$

(3) $$D=\sqrt{hh\times {\left(x_{c}-x_{c}^{gt}\right)}^{2}+ww\times {\left(y_{c}-y_{c}^{gt}\right)}^{2}+B}$$

(4) $$NWD^{shape}=e^{-\frac{D}{C}}$$

Here, B is the size penalty term (w-w_gt_)^2^+(h-h_gt_)^2^ normalized by weigh^2^, where weigh is a scale factor. The w and h refer to the width and height of the predicted box, and w^gt^ and h^gt^ correspond to the width and height of the ground-truth box. C is a constant temperature (normalization) coefficient. D denotes the shape-weighted squared distance that combines the weighted center deviations and the size penalty B. The coefficients hh and ww are shape-dependent weights for the center offsets, typically set to the average height (h-h_gt_)/2 and average width (w-w_gt_)/2. The coordinates (x_c_,y_c_) and $\left({\text{x}}_{\text{c}}^{\text{gt}},{\text{y}}_{\text{c}}^{\text{gt}}\right)$ denote the center points of the predicted bounding box and the ground-truth box, respectively.

Compared to IoU-based methods, which rely solely on overlap area, Shape-NWD offers several distinct advantages due to its unique weighted distance computation mechanism:

1. Its design inherently incorporates higher sensitivity to variations in bounding box shape and scale. This is because Shape-NWD not only considers the conventional geometric deviation when defining the distance metric D, but also introduces the Shape correlation coefficient h_h_ and w_w_, as well as the width and height difference term B. This design allows it to accurately characterize the morphological variation of the bounding box, for example, to distinguish the different impact on localization error of slender bar objects and square objects.
2. Through the exponential normalization function e^−D/C^, this loss maps the original distance to a smooth interval of [0, 1], which enhances the training stability. This transformation effectively avoids the problem of gradient fluctuation or disappearance that may occur when the distance value is too large or too small.

## 3 Experimental results and analysis

### 3.1 Experimental configuration

The experimental configuration of this study uses the following hardware and software combinations: A computer system running Windows 11 with an NVIDIA RTX 5090 graphics card (32 GB VRAM), an Intel Core U9-285K processor, 64GB of RAM (Kingston, frequency 6000), and a 3TB solid state drive. The software environment includes Python 3.11.5, Anaconda 3, CUDA 12.8, CuDNN 9.8.0, and PyTorch 2.8.0.

According to the hardware configuration and model parameters, the batch size is set to 512, the initial learning rate is 0.01, and the momentum factor, weight decay and training rounds are configured to 0.9, 0.0005 and 100, respectively. The training data set is a self-built strawberry phenology image data set, and the input images are uniformly adjusted to 640×640 pixels. The Shape-NWD loss weights are α=0.5, β=0.5, determined through validation-set grid search. Confidence and IoU thresholds for post-processing: 0.25 and 0.7, respectively.

Statistical Validation Protocol. To ensure the reliability and reproducibility of our experimental results, all primary detection performance metrics are evaluated using 5-fold cross-validation. The dataset is randomly partitioned into five folds with stratified sampling to maintain class distribution. Each model is trained and evaluated five times, with each fold serving as the test set once. The final performance is reported as mean ± standard deviation over the five runs. This protocol is applied uniformly to both the ablation studies (Section 3.6) and the comprehensive comparison with state-of-the-art models (Section 3.7).

### 3.2 Experiment on cross-level visual feature fusion

This experiment aimed to solve the problem of insufficient cross-level and cross-location information interaction in SOD. Based on HyperC2Net, the hypergraph calculation was used to capture complex high-order correlations, so as to synthesize cross-level visual features. The Hyper-YOLO architecture, incorporating hypergraph convolution, was integrated into the neck network. The EfficientViM neck module for channel information mixing, Gold-YOLO and ASF-YOLO (Kang et al., 2024) for strengthening SOD ability, HSFPN structure (Chen et al., 2024) for cross-scale fusion strategy, GFPN structure and WFU module (Li et al., 2024), and SEAM module for enhancing the local feature representation ability of small targets were selected. The results are detailed in **Table 4**.

**Table 4:** Experimental results of neck feature networks of different models.

Model	P (%)	R (%)	F1 (%)	mAP (%)	Model Size (MB)	FLOPs (G)	Params (M)
YOLOv12n	85.4	86.0	86	90.8	5.4	6.0	2.52
+ EfficientViMBlock	84.4	85.7	85	90.3	6.3	19.3	2.96
+ Gold-YOLO	88.2	87.1	88	92.2	11.7	10.5	5.62
+ HSFPN	86.2	85.2	85	90.7	4.1	6.5	1.87
+ WFU	85.2	84.3	85	89.6	7.6	8.3	3.59
+ ASF_YOLO	86.5	85.5	86	91.5	6.2	7.9	2.88
+ SEAM	85.9	88.3	87	92	5.7	6.6	2.65
+ GFPN	86.8	86.5	87	91.3	6.6	7.5	3.11
+ Hyper_YOLO	87.9	87.7	88	92.3	6.8	8.2	3.17

**Figure 4:**
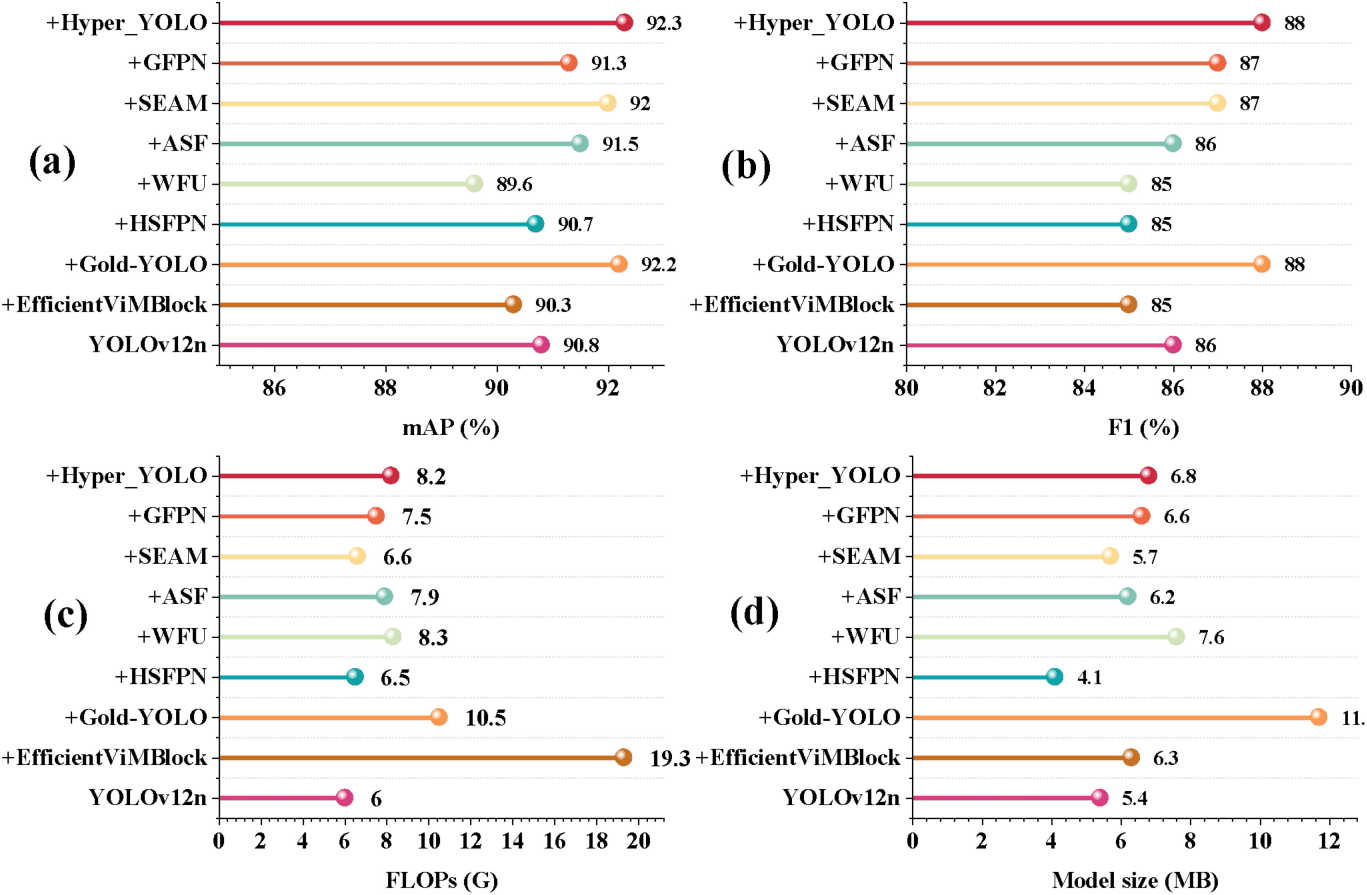
Comparison of precision and complexity of feature fusion experiments. **(a)** mAP. **(b)** F1. **(c)** FLOPs. **(d)** Model Size.

As shown in the table above, the introduction of the EfficientViMBlock and WFU module results in inferior performance across all metrics for both accuracy and complexity compared to the baseline. The possible reason is that the deep information is lost during node propagation. Compared to the baseline model, the Gold-YOLO approach demonstrated superior performance across all accuracy metrics, and the F1 score had increased by 2%, but the cost is a sharp increase in model complexity indicators. The fundamental reason is that the multi-branch aggregation under the GD mechanism leads to an increase in computational cost. In contrast, although the HSFPN has a simple feature fusion method and achieves the smallest model volume and parameter quantity, it cannot dynamically focus on useful information and suppress redundant information, resulting in a decrease in average precision compared to Gold-YOLO. Similarly, the ASF and SEAM modules, which are also aimed at optimizing the detection effect of small targets, have increased the mAP by 0.7% and 0.5% respectively, and the latter has more advantages in FLOPs and model volume. Although the F1 score of GFPN is the same as that of SEAM at 87%, its mAP is 0.7% lower, and the repeated cross-connections lead to an increase in model volume and parameter quantity, increasing by 0.9MB and 0.46M respectively. Finally, Hyper_YOLO connects the small target and context features to achieve feature enhancement and collaborative detection, reaching the peak in both mAP and F1 indicators, which are 92.3% and 0.88 respectively. Therefore, this study selected Hyper_YOLO as the neck improvement of the baseline network for strawberry phenological feature fusion. The specific improvement effect is shown in **Figure 4**.

As depicted in **Figure 4**, Hyper_YOLO achieves the highest mAP value. Compared with the baseline model, although the model size slightly increases, the mAP rises by 1.5% (**Figure 4a**). Moreover, compared with Gold-YOLO, although both models outperform YOLOv12n in terms of performance, Hyper_YOLO demonstrates a smaller model volume and FLOPs (**Figures 4c, d**), making it more suitable for lightweight deployment. Considering the accuracy and complexity indicators of the seven improvement schemes comprehensively, the Hyper_YOLO scheme has the best detection effect and is named H-Net.

### 3.3 Attention mechanism comparison experiment

To further enhance local feature information, this study investigates the effects of the related attention mechanism on the detection of strawberry phenology. A MSCA module, which balances representational capacity and computational efficiency, was integrated into the backbone network. For comparison, six other attention mechanisms were also evaluated: the Hierarchical Reciprocal Attention Mixing (H-RAMi) module (Choi et al., 2024) and the Multidimensional Collaborative Attention (MCA) module (Dai et al., 2026), both designed for multi-scale feature details; the Parallelized Patch-aware Attention (PPA) module (Xu et al., 2024) for increased focus on small objects; the Convolution and Attention Fusion Module (CAFM) (Hu et al., 2024) combining convolutional and self-attention operations; and the Fine-grained Channel Attention (FCA) module alongside the Normalization-based Attention Module (NAM) (Sun et al., 2024) for local and global information interaction. The specific experimental results are detailed in **Table 5**:

**Table 5:** Experimental results of different attention mechanism comparison.

Model	P(%)	R(%)	F1(%)	mAP(%)	mAP@.5:.95(%)	FLOPs (G)	Params (M)	Size (MB)
H-Net	87.9	87.7	88	92.3	65.9	8.2	3.17	6.8
+ MSCA	89.9	88.9	89	94.6	71.9	8.9	3.17	6.7
+ H-RAMi	88.0	87.0	87	92.5	65.9	8.3	2.69	5.6
+ PPA	90.3	89.0	89	94.3	71.4	10.6	5.26	10.9
+ MCA	89.0	90.1	89	94.4	71.5	9.1	3.22	8.9
+ CAFM	89.2	89.5	89	94.1	70.8	9.3	3.44	7.3
+ FCA	90.1	89.3	89	94.2	71.6	9.1	3.16	6.7
+ NAM	89.1	90.1	89	94.6	71.3	9.0	3.09	6.6

**Figure 5:**
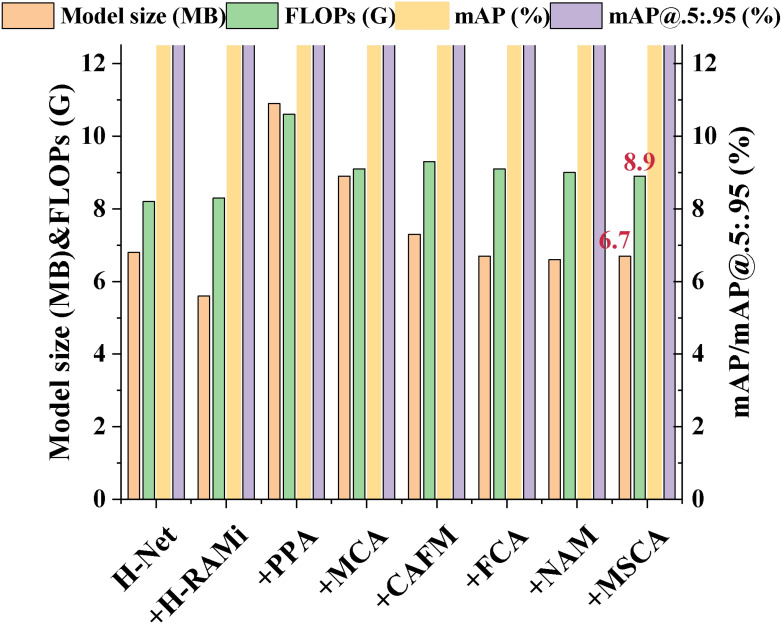
Comparison of attention mechanism effects.

As listed in **Table 5**, after introducing the H-RAMi module, the F1 score decreases by 1%, due to the attention weight of the SOD problem, which is susceptible to noise. The MCA module, which is also aimed at multi-scale features, constructs the interaction between two parallel axial attention, which effectively enhances the weight utilization and the detection effect. The introduction of MSCA further improved the detection effect, and the spatial adaptive feature selection and enhancement were realized by weighting the features of different positions, and the mAP value and mAP@.5:.95 value both reached the peak. At the same time, compared with MSCA, although NAM has similar detection accuracy, the channel-by-channel scaling technology affects the detection speed, and the indicators of CAFM and FCA are weaker than MSCA. Although the PPA module delivers moderate detection accuracy, its multi-scale pooling operations involve repeated sampling, which significantly increases model complexity. See **Figure 5** for a comparison of each attention mechanism.

According to **Figure 5**, the introduction of MSCA module on the basis of H-Net can multi-scale feature extraction without substantially increasing computational complexity, thus contributing to the accurate identification of strawberry phenology. The improved scheme reaches a peak of 71.9% on the mAP@.5:.95 accuracy index, which is named HM-Net.

### 3.4 Performance impact of different detection heads

To enhance the sensitivity to subtle changes in early phenology, the detection performance of strawberry phenology stage was improved by replacing different detection heads. In this experiment, two high-precision detection heads: Adaptively Spatial Feature Fusion(ASFF) and D_PPA are selected. And three lightweight detection heads Mobile Bottleneck Convolution (MBConv), Conv2Former and Detect dynamic Head (DyHead) (Han et al., 2024). Among them, ASFF effectively utilizes multi-scale information through weight adaptive fusion, while PPA forms a SOD head with attention. DyHead, MBConv and Conv2Former rely on dynamic weight fusion, large kernel convolution operation and moving inversion bottleneck structure to achieve lightweight detection, respectively. The detection head improvement data are shown in **Table 6**.

**Table 6:** Data from each detection head improvement.

Model	P (%)	R (%)	F1(%)	mAP (%)	mAP@.5:.95(%)	Size (MB)	FLOPs(G)	Params (M)
HM-Net	89.9	88.9	89	94.6	71.9	6.7	8.9	3.17
+ ASFF	89.7	89.4	89	94.7	72.8	10.2	12.5	4.88
+ Conv2Former	89.5	90.4	90	94.6	70.7	6.4	8.4	2.99
+ DyHead	89.7	90.0	90	94.0	70.7	6.3	7.6	2.98
+ MBConv	90	89.5	89	94.5	70.2	6.1	7.8	2.86
+ D_PPA	89.1	89.7	89	94.6	70.1	8.1	12.0	3.80

**Figure 6:**
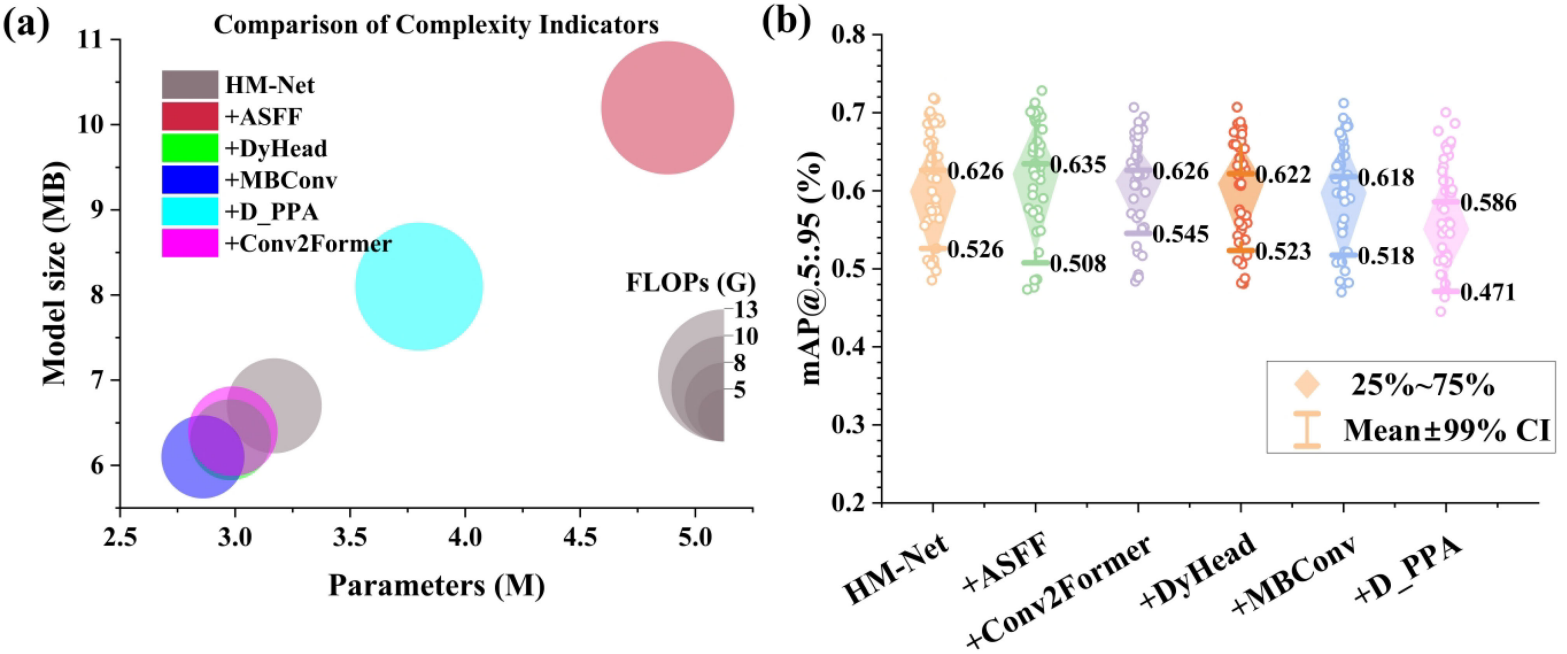
Figure of detection head test results. **(a)** Comparison of Complexity Indicators. **(b)** Comparison of Accuracy Indicators.

As summarized in **Table 6**, although the mAP value of ASFF is 0.1% higher, the model complexity index increases, which is not conducive to lightweight deployment. Similarly, D_PPA achieves the same mAP value as the baseline, but has a 1.4MB larger model size and 3.1G higher FLOPs. This suggests that the above modules may have introduced additional nonlinear or dynamic weights, making the inherently sensitive end of the detection head more difficult to optimize. Although the computational complexity of DyHead is smaller than that of HM-Net, the mAP is reduced by 0.6 percentage points, which may be because the learning of attention weight in the feature fusion does not converge to the optimal solution, thus limiting the improvement of the upper limit of accuracy. Notably, among the alternatives with lower computational cost than HM-Net, although the complexity of Conv2Former is slightly higher than MBConv, there is no accuracy loss. Therefore, Conv2Former achieves the overall optimal performance, and the visualization results presented in **Figure 6**.

As illustrated in **Figure 6a**, Conv2Former maintains a compact profile in terms of model size, parameters, and FLOPs. Combined with the data distribution shown in **Figure 6b**, it is evident that the mAP@.5:.95 values for Conv2Former exhibit the most concentrated interquartile range (25%–75%). While ASFF achieves the highest detection accuracy, its performance distribution is considerably more dispersed. Consequently, in this section, the more lightweight detection head Conv2Former is adopted without compromising accuracy, and the resulting model is designated as HCM-Net.

### 3.5 The influence of different loss functions on the model

The loss function facilitates parameter optimization during model training by quantifying the discrepancy between predictions and ground truth. In this study, various loss functions are investigated on the basis of HCM-Net to enhance strawberry phenology detection performance. A total of 13 loss functions are compared, namely CIOU, DIOU, GIOU, ATFL, SD Loss, Shape-IOU, Shape-NWD, Focaler-DIoU, Focaler-GIoU, Focaler-CIoU, WIOU, NWD-IOU, and Slideloss. Since the choice of loss function has negligible impact on model complexity, only the accuracy outcomes are reported, as summarized in **Table 7**.

**Table 7:** Experimental results of different loss function comparison.

Model	P(%)	R(%)	F1(%)	mAP(%)	mAP@.5:.95(%)
HCM-Net(CIOU)	89.5	90.4	90	94.6	70.7
+ DIOU	90.3	88.7	89	94.3	70.0
+ GIOU	90.8	88.5	89	94.3	70.8
+ ATFL	89.7	89.1	89	94.4	71
+ SD Loss	90.0	89.2	89	94.1	69.5
+ Shape-IOU	91.2	88.4	90	94.7	69.5
+ Shape-NWD	90.5	89.8	90	94.9	71.9
+ Focaler-DIoU	89.6	88.6	89	94.3	70.5
+ Focaler-GIoU	89.0	90.1	89	94.5	70.5
+ Focaler-CIoU	88.3	89.9	89	94.6	70.8
+ WIOU	90.8	89.3	90	94.6	70.7
+ NWD-IOU	89.8	89.5	89	94.5	70.4
+ Slideloss	88.8	90.1	89	94.2	70.1

As presented in the table, Shape-NWD demonstrates superior performance, achieving peak values of 94.9% in mAP and 71.9% in mAP@.5:.95, significantly surpassing all other loss functions. Furthermore, for small objects, the fixed window strategy employed by Slideloss may fail to accurately capture their geometric characteristics, leading to distorted matching measurements. The reason for the poor performance of SD Loss may be that the feature distribution of small targets is sparse and noise-sensitive, and SD Loss based on statistical measures is more susceptible to feature fluctuations. The three Loss functions Focaler-DIoU, Focaler-GIoU and Focaler-CIoU are based on the classic IoU variant and introduce the idea of Focal Loss to make the model more focused on the prediction boxes that are not aligned or have low IOU values. However, this focused set mechanism is just adjusting the weights of different samples. No matter how weighted, they still rely on traditional geometric measures such as DIoU and GIoU. In contrast, traditional loss functions such as DIoU and GIoU, although they are OK on some individual indicators, the overall improvement is not outstanding, mainly because they have limited gradient information for non-overlapping cases and are not sensitive to shape and scale.

**Figure 7:**
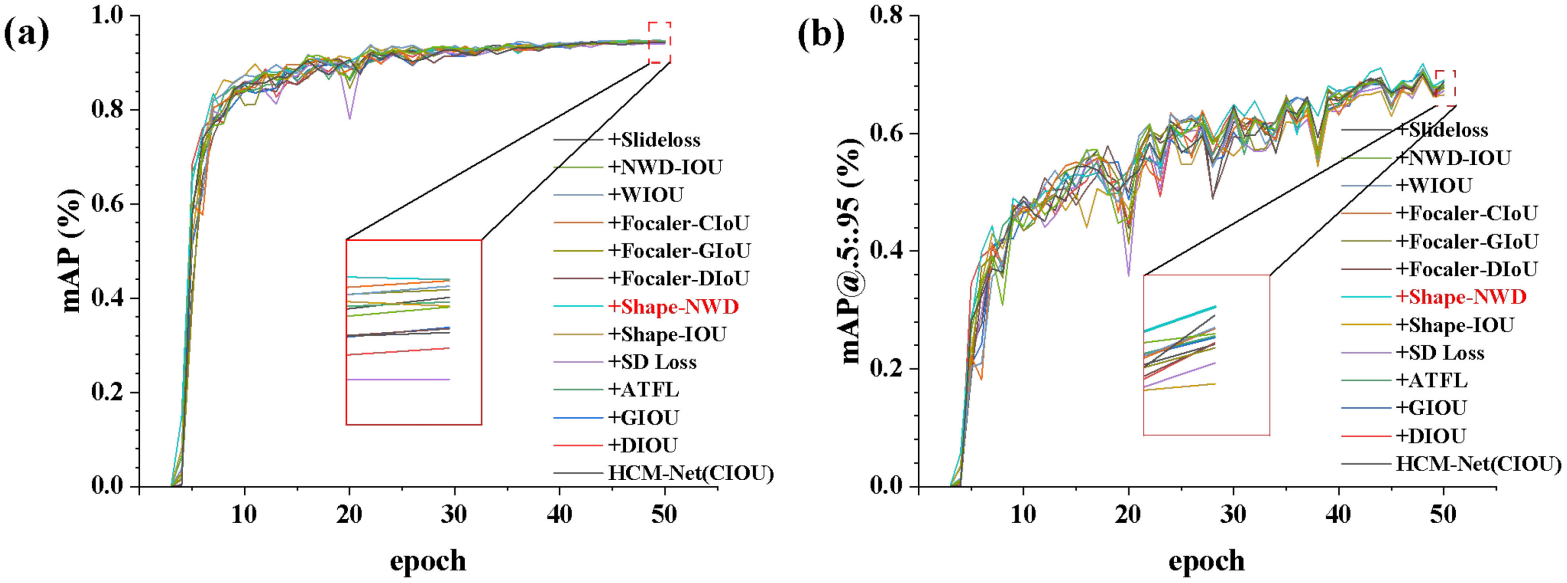
Comparison of results with different loss functions. **(a)** Comparison of mAP. **(b)** Comparison of mAP@.5:.95.

We applied the same gradient analysis to other top-performing losses (e.g., Focaler-CIoU, WIOU) and found that their gradient fields either retain the non-overlapping dead zone (CIoU variants) or lack explicit shape constraints (WIOU). Shape-NWD is thus theoretically justified as the optimal choice, and the 13-loss benchmark experiments serve as the empirical validation of this theoretical prediction. See **Figure 7** for the visualization of the accuracy of each loss function.

Conventional loss functions often encounter performance plateaus when dealing with small objects and multi-scale detection tasks. Shape-IoU, which is specifically designed for shapes, is a bit less effective, despite its name. In contrast, Shape-NWD diverges from the traditional IoU-based paradigm by adopting the NWD, which measures the similarity between Gaussian distributions associated with bounding boxes. The experimental results confirm its optimal performance, as indicated by the red markers in **Figure 7**. By comparing the mAP and mAP@.5:.95 indicators of different loss functions in the training process, it can be clearly seen that Shape-NWD can better fit the training data, showing a strong momentum.

### 3.6 Ablation experiment results and discussion

#### 3.6.1 Ablation experiment

In order to comprehensively demonstrate the influence of key modules and optimization strategies, this study designed ablation experiments for systematic evaluation based on HyperC2Net and YOLOv12n models, and the detailed data are summarized in **Table 8**.

**Table 8:** Results of ablation experiments.

Module	Hyper	MSCA	Conv2Former	Shape-NWD	mAP(%)	mAP@.5:.95(%)	F1 (%)	Params (M)	FLOPs (G)	Size (MB)
H-Net	✓	×	×	×	92.3 ± 0.3	65.9 ± 0.4	88	3.17	8.2	6.8
HM-Net	✓	✓	×	×	94.6 ± 0.2	71.9 ± 0.3	89	3.17	8.9	6.7
HC-Net	✓	×	✓	×	93.3 ± 0.5	67.3 ± 0.4	88	2.98	7.7	6.5
HS-Net	✓	×	×	✓	93.4 ± 0.5	67.1 ± 0.6	88	3.17	8.2	6.8
HCM-Net	✓	✓	✓	×	94.6 ± 0.4	70.7 ± 0.5	90	2.99	8.4	6.4
HCS-Net	✓	×	✓	✓	94.1 ± 0.4	69.5 ± 0.6	89	2.98	7.7	6.5
HMS-Net	✓	✓	×	✓	94.0 ± 0.3	70.7 ± 0.5	89	3.17	8.9	6.7
HCMS-Net	✓	✓	✓	✓	94.9 ± 0.2	71.9 ± 0.3	90	2.99	8.4	6.4

As illustrated in **Table 8**, the consistent small standard deviations demonstrate the high stability of the proposed modules. The full HCMS-Net achieves a 2.6 percentage point increase in mAP@0.5 with a tiny standard deviation of ±0.2%, indicating robust performance across different data splits. Moreover, integrating the HyperC2Net with the YOLOv12n neck yielded an increase of 1.5% in mAP and 2% in F1-score. Secondly, after the introduction of MSCA attention mechanism, F1, mAP and mAP@.5:.95 increased by 1%, 2.3% and 6%, respectively. Then, after integrating the lightweight Conv2Former module into the detection head, the mAP of HCM-Net remains unchanged, and the F1 score was slightly improved. Meanwhile, the number of model parameters, GFLOPs, and model size were reduced by 5.7%,5.6%, and 4.5% respectively. This is because the long-range context from the lightweight Conv2Former dynamically guides the local attention of MSCA.

Finally, the HCMS-Net model was successfully constructed by replacing the original loss function with Shape-NWD. The model complexity did not change, but mAP@.5:.95 reached 71.9%, marking a 1.2 percentage point increase. When applied individually, each module encounters a performance bottleneck: Conv2Former alone (+1.0%) enriches long-range contextual features, but its refined representations cannot be fully exploited due to the coarse localization metric (standard IoU loss), which is insensitive to the shape and scale of tiny strawberries. Shape-NWD alone (+1.1%) provides a better optimization objective for small objects, but its effectiveness is limited by the residual background noise in the feature map, as the backbone has not been specifically enhanced to suppress it. When integrated, the two components create a mutually reinforcing loop. Conv2Former suppresses complex background clutter and provides cleaner, boundary-aware feature maps. This high-quality input allows Shape-NWD to precisely supervise the fine-grained geometry of the tiny targets. In return, the precise gradients from Shape-NWD further sharpen the attention of Conv2Former at the boundary regions. This unlocks a performance ceiling that neither module could reach alone. Notably, the combined gain (1.8%) is not a simple arithmetic sum of individual gains (1.0% + 1.1%), indicating a specific, non-linear coupling rather than independent, additive effects. This strongly validates the architectural coherence of our design. The detection outcomes of each improved variant across different phenological stages are detailed in **Table 9**.

**Table 9:** Results of ablation experiment for each phenological period.

Model	AP(%)					P (%)	R (%)
	Flower	Early	Green	Turning	Ripe		
YOLOv12n	98.3	80.8	84.5	93.1	97.4	85.4	86.0
H-Net	99.0	83.3	86.7	95.1	97.3	87.9	87.7
HM-Net	99.3	86.8	91.5	96.9	98.5	89.9	88.9
HCM-Net	99.2	87.5	91.5	96.5	98.1	89.5	90.4
HCMS-Net	99.3	88.3	90.9	97.0	98.2	90.5	89.8

**Figure 8:**
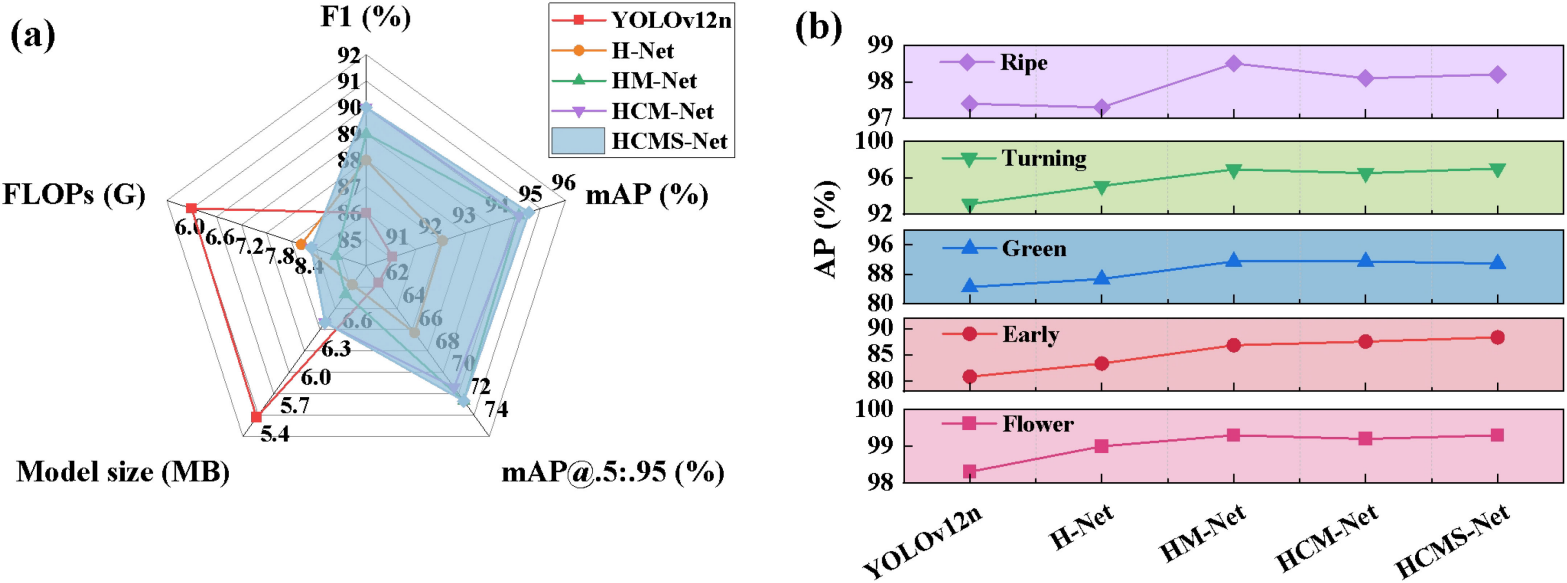
Visualization of ablation experiments. **(a)** Results of ablation experiments. **(b)** Detection results at different phenological stages.

The AP index of H-Net at the flowering stage increased by 0.7% compared to the baseline, and mAP@.5:.95 increased by 4.4 percentage points. In the green fruit stage, the AP value of HM-Net increased by 4.8% on the basis of H-Net, and the effect was significant. Although the AP value of HCMS-Net was 0.6% lower than that of HCM-Net at the green fruit stage, the other indicators were higher than that of HCM-Net on the whole. It is verified that the Shape-NWD loss function provides a more stable and discriminative gradient optimization signal for SOD after introducing the morphological related weighting term and the scale adaptive distance metric. See **Figure 8** for a visualization of the complete data.

It can be seen from **Figure 8a** that HCMS-Net shows a relatively balanced performance, especially in the accuracy index. **Figure 8b** shows the comparison of AP index of each phenological stage on different models. After each improvement, the AP value of each phenological stage fluctuated slightly, but showed an increasing trend as a whole, further indicating that the comprehensive performance of HCMS-Net has more advantages in strawberry phenological stage recognition.

#### 3.6.2 Visualized analysis

In order to more intuitively understand the attention of different improvement modules in the model to strawberry feature information, especially for such tasks with continuous morphological changes, we decided to use HiRes-CAM heat maps for visual analysis. In this way, it is more helpful to explore which parts of the thermal image the model focuses on, such as whether the model focuses on the expansion of the strawberry petal or the adjacent stem and leaf, and whether the model focuses on the edge of the strawberry fruit or the fruit itself. Heatmaps from key phenological stages are compared collectively to examine differences in model behavior across stages.

**Figure 9:**
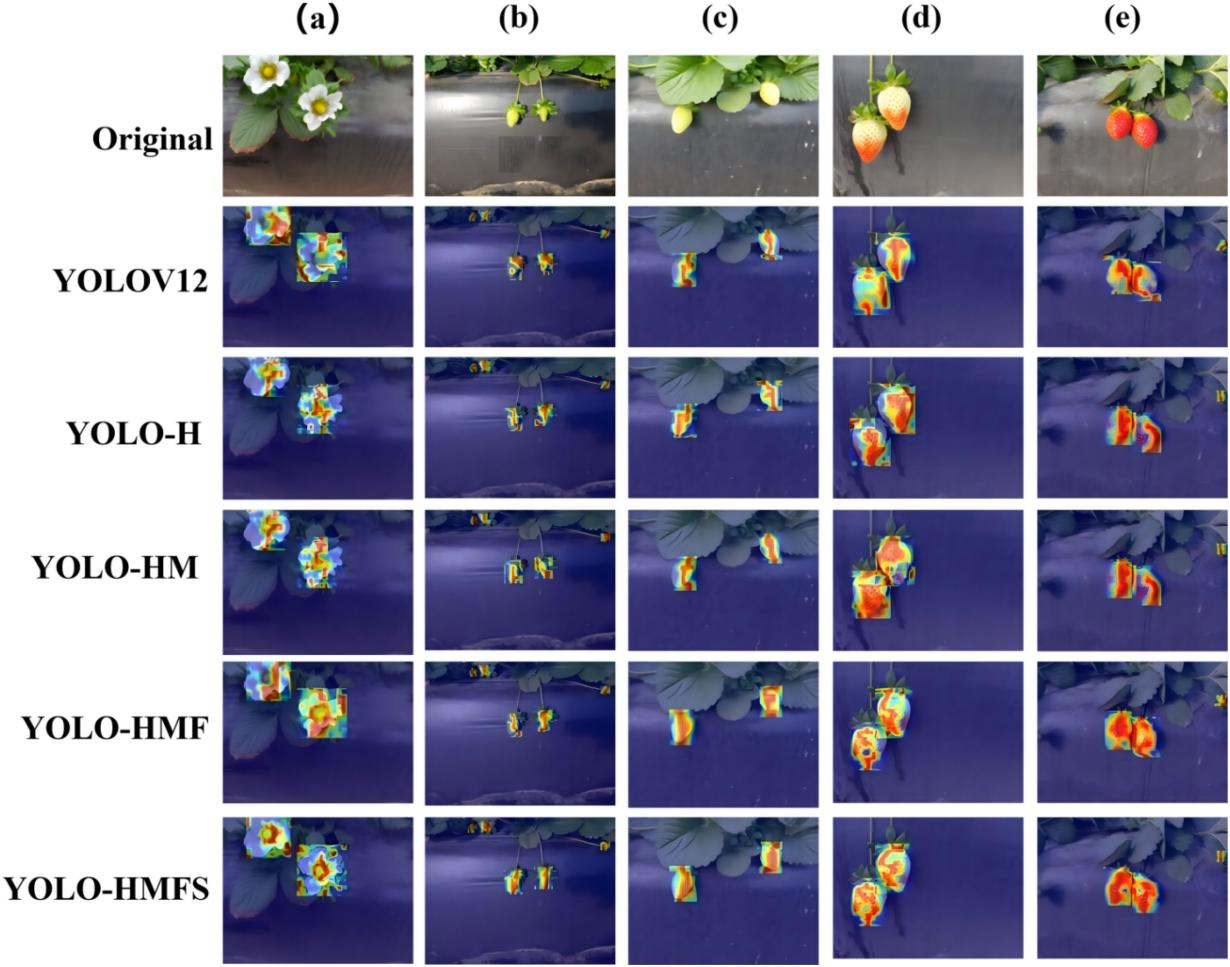
Comparison of thermodynamic maps in ablation experiments. **(a)** Flower **(b)** Early **(c)** Green **(d)** Turning **(e)** Ripe.

To move beyond qualitative observation, we perform a hypothesis-driven analysis of the generated heatmaps. In strawberry phenology recognition, we hypothesize that the most discriminative visual cues are the sharp color transitions between green and red patches on the fruit surface, and the texture boundaries formed by the characteristic raised areas of the peel at different maturity stages. If HCMS-Net truly learns phenology-aware features, its activation maps should precisely localize these boundaries rather than simply fixating on the fruit centroid or background foliage. The heatmap comparisons from the ablation study are presented in **Figure 9**.

The heatmaps in **Figure 9** provide qualitative support for this hypothesis. For a “Early” and “Green” fruit, HCMS-Net’s strongest activations (highlighted in red) tend to concentrate along the annular margin where the green calyx meets the whitening pericarp, a visually salient region that is visually diagnostic for this specific phenological transition. In contrast, the baseline model YOLOv12n displays diffuse, high-intensity activation across the entire fruit body and surrounding leaves, without clearly isolating this discriminative boundary. For ripe fruits, HCMS-Net often consistently focuses on the high-curvature textured protrusions on the peel, whose density and roughness are associated with maturity. YOLOv12n, however, is more frequently activated by co-occurring background elements like stems or shadows.

As illustrated in **Figure 9**, the activated regions in the heatmaps of H-Net are largely concentrated within the contour of the strawberry fruit, whereas those of YOLOv12n spread noticeably toward the fruit edges. Starting from the young-fruit stage, the model’s activated regions begin from local parts of the fruit and gradually expand to the entire fruit as maturity increases. This pattern suggests that the network’s attention may capture the progression of color change to some degree, rather than relying solely on static pattern matching. Among the compared models, HCMS−Net exhibits a more focused attention distribution across all five key phenological stages. For example, at the flowering stage, HCMS−Net attends more precisely to the flower bud than the other improved networks, whose activated regions remain relatively scattered. This observation is consistent with the intended function of the Shape−NWD loss, whose shape−aware term encourages the model to explicitly utilize boundary geometric information. In addition, in the detection of the young-fruit, green-fruit, color-turning, and mature-fruit stages, the thermal activation region of HCMS−Net more tightly follow the fruit contour, while those of the baseline model are more dispersed. This improvement can be attributed to the MSCA model, which is designed to suppress background responses that are semantically unrelated to the target.

We note that these HiRes-CAM visualizations are derived from a single representative layer and should be regarded as complementary qualitative evidence, rather than a complete mechanistic explanation. A more comprehensive understanding would require layer-wise analysis across multiple network depths, as demonstrated in the palm detection study by (Cui et al., 2025). Such multi-layer examination is planned for future work to further validate the hierarchical feature refinement process within HCMS-Net.

#### 3.6.3 Synergistic mechanism of HCMS-Net

The ablation study reveals a defining property of HCMS-Net: individual modules produce non-additive gains, indicating architectural synergy rather than simple stacking. The non-additive gain of Conv2Former + Shape−NWD (+1.0% and +1.1% individually → +1.8% jointly) is direct evidence of architectural synergy. We explain this by a “feature–optimization” closed loop: Conv2Former denoises feature maps via global context, allowing Shape−NWD to compute stable distributional gradients even for tiny targets; these precise gradients in turn sharpen Conv2Former’s attention toward surviving object fragments. This bidirectional reinforcement, validated by gradient field visualizations and consistent across other module pairs (e.g., MSCA + HyperC2Net), proves that HCMS−Net behaves as a coherent architectural system, not a modular patchwork.

The observed non-additive gain from combining Conv2Former and Shape-NWD (+1.8% mAP) can be explained by their gradient-level interaction. The gradient of Shape-NWD with respect to the feature map is proportional to the spatial attention weight from Conv2Former. When the feature map contains less background activation (due to Conv2Former), Shape-NWD’s gradient more precisely targets the object boundaries, creating a mutually reinforcing optimization loop.

### 3.7 Comparative experiment on mainstream networks

To demonstrate the advantages of our method over others, this experiment benchmarks with multiple one-stage mainstream networks, mainly including nano-volume lightweight YOLO models (YOLOv3tiny, YOLOv5n, YOLOv8n, YOLOv9tiny, YOLOv10n-13n), RT-DETR-R18, LUD-YOLO, and SOD-YOLO models.

To ensure rigorous fairness, all compared detectors are retrained and evaluated under completely identical settings on our self-built strawberry dataset. The exact same training, validation, and test splits are used for every model. All models share a unified training recipe, including input size of 640×640, SGD optimizer, base learning rate, cosine annealing schedule, weight decay, batch size, and a total of 100 training epochs. No model-specific augmentation tricks are applied beyond what is inherent to the standard architecture. All hyperparameters are kept strictly the same across models, and convergence is verified for each model by monitoring validation loss curves. **Table 10** compares the performance of SOTA detectors using a 5-fold cross-validation method (mean ± std).

**Table 10:** Comparison of mainstream algorithms.

Model	F1(%)	mAP(%)	mAP@.5:.95(%)	Size (MB)	FLOPs(G)	Params(M)
RT-DETR-R18	88	91.8 ± 0.4	66.4 ± 0.5	66.2	60.0	32.81
YOLOv3tiny	87	91.5 ± 0.6	63.7 ± 0.8	24.4	19.0	12.13
LUD-YOLO	86	87.8 ± 0.5	63.5 ± 0.6	5.6	9.2	2.81
SOD-YOLO	84	85.1 ± 0.7	64.1 ± 0.8	3.3	4.1	1.57
YOLOv5n	85	90.7 ± 0.6	61.9 ± 0.7	5.2	7.2	2.51
YOLOv8n	86	91.5 ± 0.4	65.7 ± 0.5	6.2	8.2	3.01
YOLOv9tiny	81	86.9 ± 0.4	60.4 ± 0.6	4.6	7.9	2.01
YOLOv10n	86	90.7 ± 0.5	63.5 ± 0.7	5.7	8.4	2.71
YOLOv11n	86	90.3 ± 0.6	62.5 ± 0.7	5.4	6.4	2.59
YOLOv12n	86	90.8 ± 0.4	61.5 ± 0.5	5.4	6	2.52
YOLOv13n	84	88.9 ± 0.4	58.5 ± 0.6	5.4	6.4	2.46
YOLOv12s	88	92.2 ± 0.3	67.1 ± 0.5	18.6	19.6	9.10
H-Net	88	92.3 ± 0.3	65.9 ± 0.4	6.8	8.2	3.17
Ours	90	94.9 ± 0.3	71.9 ± 0.4	6.4	8.4	2.99

**Figure 10:**
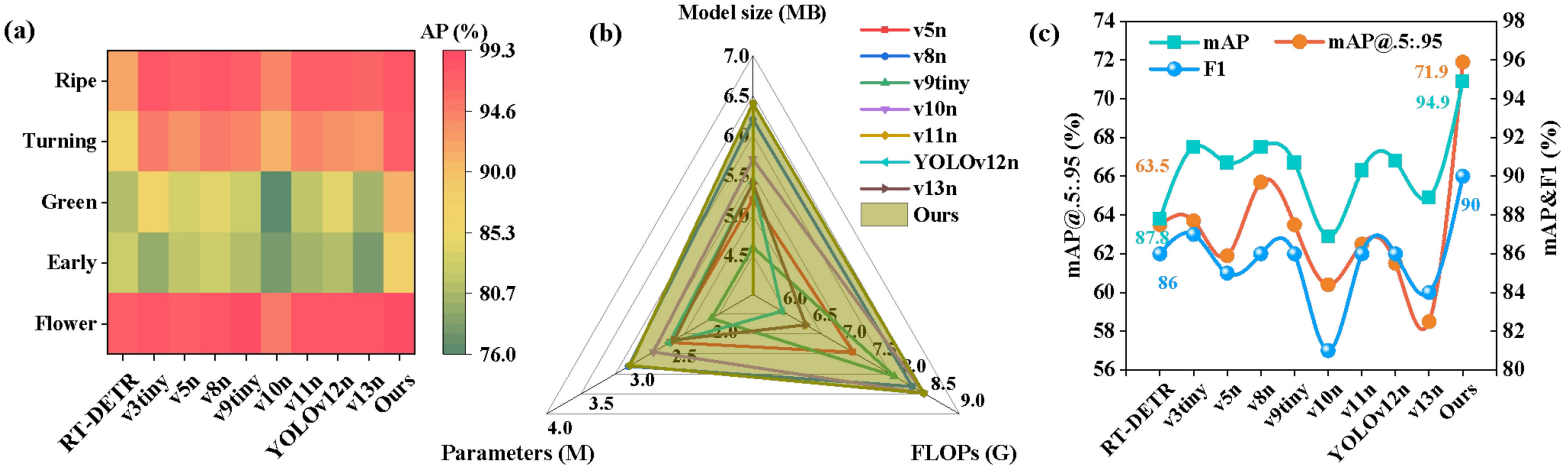
Experimental results of different mainstream networks. **(a)** Experimental results of different phenological periods. **(b)** Complexity comparison results. **(c)** Precision comparison results.

HCMS-Net achieves a mean mAP@0.5 of 94.9% with a standard deviation of only ±0.3%, demonstrating both superior accuracy and remarkable stability across different data splits. It confirms that the performance margin is statistically highly significant and not an artifact of a particular data partition. This is further corroborated by the mAP@0.5:0.95 metric, where HCMS-Net’s 71.9% ± 0.4% significantly surpasses YOLOv12n’s 61.5% ± 0.5%. To further illustrate the stability across folds, we provide the per-fold mAP and mAP@.5:.95 traces for HCMS-Net and YOLOv12n in **Figure 10c**. The curves show that HCMS-Net consistently outperforms the baseline in every single fold, with no overlap in performance distribution, eliminating any doubt about the fairness of the comparison.

Compared with YOLOv12n network, the F1 score of our model is increased by 4 percentage points, and the mAP value is increased by 4.1%. The most significant increase is mAP@.5:.95, which is 10.4% higher than the baseline. In addition, the complexity of RT-DETR-R18 and YOLOv3tiny models is much higher than that of other comparison networks, which is not suitable for lightweight deployment. In terms of accuracy, our model surpasses RT-DETR-R18, YOLOv3tiny, YOLOv5n, YOLOv8n, YOLOv9tiny, YOLOv10n, YOLOv11n, YOLOv13n, LUD-YOLO, and SOD-YOLO by 3.1%, 3.4%, 4.2%, 3.4%, 4.2%, 8.0%, 4.6%, 5.9%, 7.1% and 9.2% in mAP, respectively, while also delivering substantial improvements in F1-score and mAP@.5:.95. The performance metrics detailed in the data in the above table clearly show that our method takes the lead in the precision-complexity tradeoff, and the data for each phenological stage are provided in **Table 11**.

**Table 11:** Results of experiments in different phenological stages.

Model	AP(%)					P (%)	R (%)
	Flower	Early	Green	Turning	Ripe		
RT-DETR-R18	98.3	79.3	86.7	95.3	97.9	88.2	86.4
YOLOv3tiny	97.2	82.3	81.2	86.2	92.1	84.7	87.4
YOLOv5n	97.6	81.7	83.2	93.8	97.4	86.0	85.2
YOLOv8n	98.2	82.6	83.8	95.1	98.0	86.5	86.0
YOLOv9tiny	98.6	81.4	82.8	94.1	97.6	86.0	85.3
YOLOv10n	95.2	77.6	76.1	90.9	94.5	81.1	81.2
YOLOv11n	98.0	80.2	82.0	94.0	97.2	87.0	85.4
YOLOv12n	98.3	80.8	84.5	93.1	97.4	85.4	86.0
YOLOv13n	97.8	77.7	80.5	92.4	96.3	85.0	82.7
YOLOv12s	99.0	84.2	85.0	95.3	97.6	87.7	87.5
Ours	99.3	88.3	90.9	97.0	98.2	90.5	89.8

**Table 11** summarizes that the AP value of our model reaches 99.3%, 88.3%, 90.9%, 97.0% and 98.2% respectively in the flowering, young-fruit, green-fruit, color-turning, and mature-fruit stages, which are significantly better than other mainstream models. Meanwhile, our model also reaches the peak in precision and recall, which are 90.5% and 89.8%, respectively. It is worth noting that among the five phenological stages, the AP index of our model is lower than 90% only in the young fruit stage. According to the analysis, some flowers and fruits coexist in this stage, polluting the local feature information and increasing the false detection rate. See **Figure 10** for the detailed visualization results.

**Figure 11:**
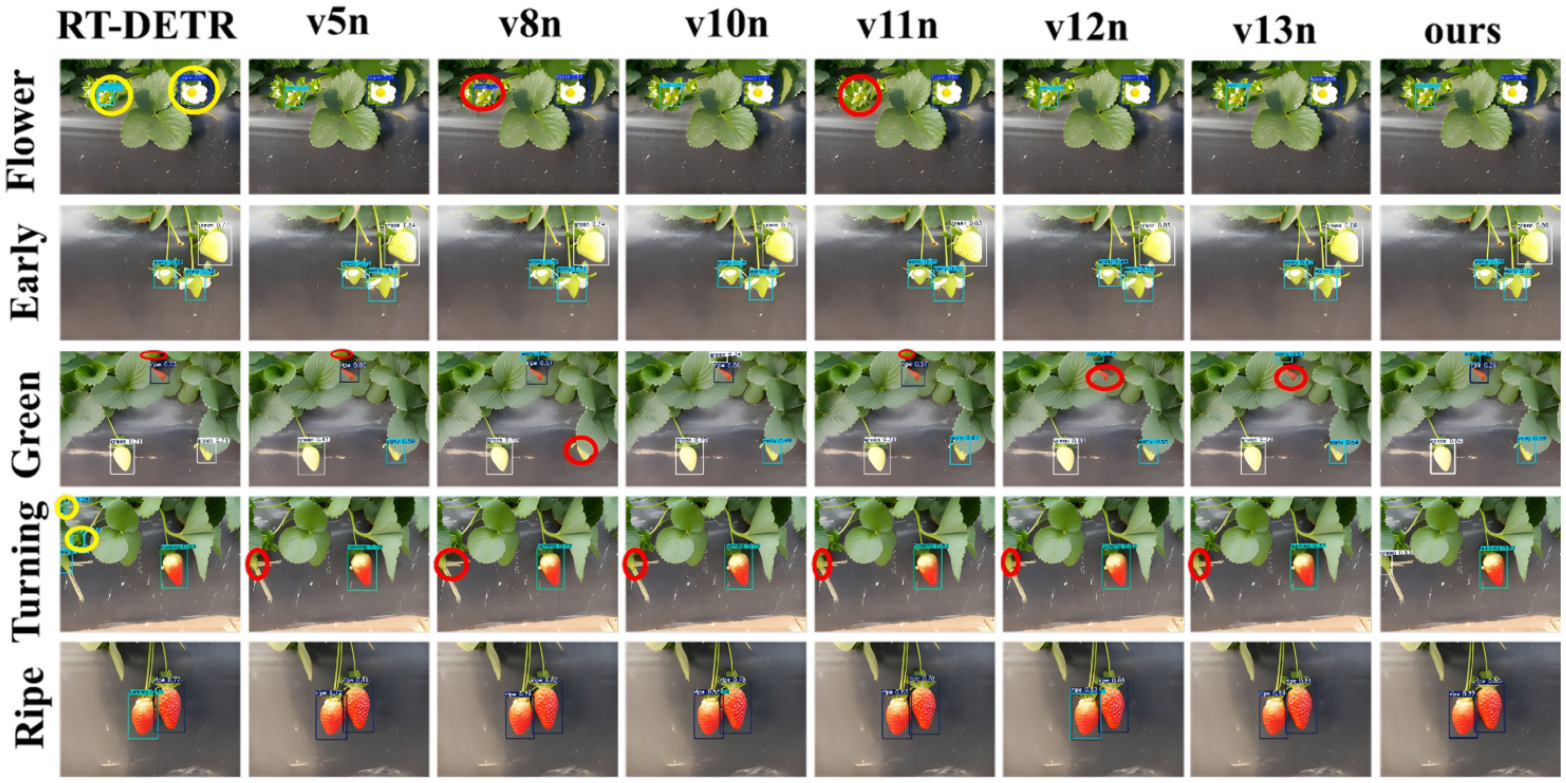
Comparison of detection performance across different mainstream networks. Red circles: missed detections. Yellow circles: false positives.

As shown in the heatmaps in **Figure 10a**, our model stands out in the identification of strawberry phenology compared with other mainstream models. Notably, the AP of each model is higher in the flowering stage, which is because at this stage, the phenological characteristics of flowers are more obvious, and the model is easier to identify. However, **Figure 10b** indicates that the model complexity still leaves space for further improvement. Combined with **Figure 10c**, we can clearly see the advantages of the improved model, and each accuracy index is higher than other models. In addition, this experiment compares the detection effect of each mainstream network on five phenological stages, and the results are illustrated in **Figure 11**.

To intuitively show the performance of HCMS-Net, we evaluate the detection effect of 8 object detection models on the test set. We can see that RT-DETR will have the problem of multiple prediction boxes for one target, which may be caused by repeated detection caused by the high NMS threshold in the post-processing. It is worth noting that in the complex field environment, most of the models missed the detection of fruits in the young fruit stage, which exactly confirms the conclusion in **Table 10** that the accuracy of the young fruit stage is relatively low. In conclusion, the HCMS-Net model proposed in this experiment has better overall detection performance for strawberry phenological stages.

### 3.8 Error analysis and discussion

To provide a balanced and transparent assessment of HCMS-Net, we systematically analyze the model’s misclassifications and detection failures. This section presents a confusion matrix for fine-grained phenological stage classification and a curated set of representative failure cases.

**Figure 12:**
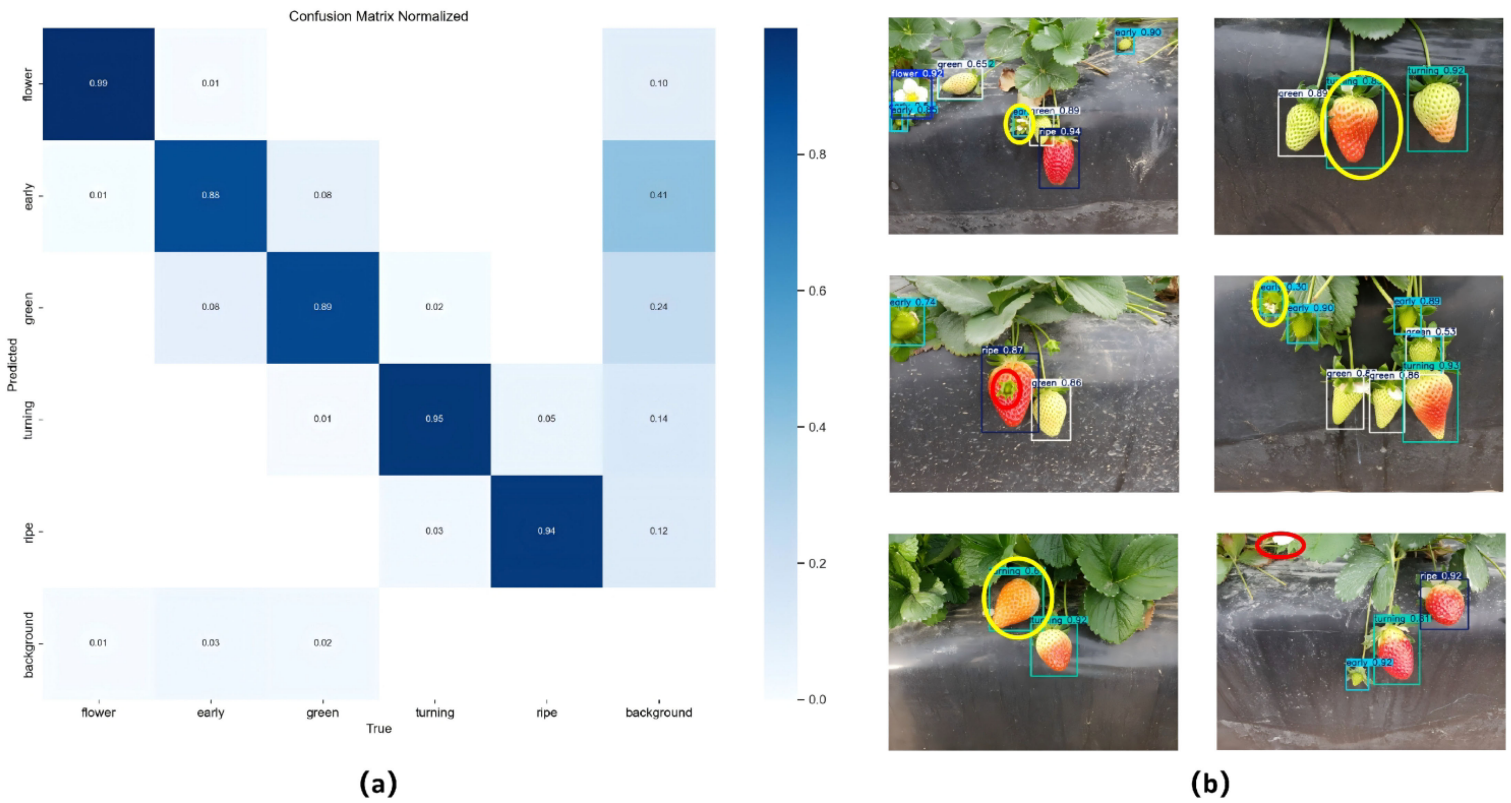
Error analysis. **(a)** Confusion Matrix. **(b)** Failure Case Analysis. Red circles: missed detections. Yellow circles: false positives.

The row-normalized confusion matrix in **Figure 12a** reveals that misclassifications are predominantly concentrated between phenologically adjacent stages. Specifically, 8% of Green-fruit samples were misclassified as Early-fruit, and 5% of Ripe-ruit samples were misclassified as Turning-fruit. Non-adjacent stages (e.g., Budding vs. Mature-fruit) exhibit near-zero confusion. This pattern is biologically expected, as adjacent stages represent a continuous ripening spectrum with gradual color transitions that are challenging even for trained annotators to discretize consistently. We note that the current annotation protocol discretizes a continuous process into five ordinal classes, and future work may explore ordinal regression or label distribution learning to better model the underlying continuum.

Beyond inter-class confusion, we identify representative detection failures to understand the model’s limitations in real-world field conditions. **Figure 12b** presents six failure cases categorized into two error types: Fine-grained misclassification under subtle color cues. A Color-turning fruit with only a small emerging red patch is misclassified as Green-fruit. The limited visible area of color transition, often less than 10% of the fruit surface, remains a challenge for RGB-based recognition. False negatives under heavy occlusion. Fruits occluded by dense leaf clusters are completely missed. Although HCMS-Net demonstrates improved occlusion robustness through MSCA and Conv2Former, extreme occlusion (>70%) still leads to missed detections, particularly when the occluding leaves share similar texture with the background.

These failure cases highlight the inherent difficulties of field-based strawberry phenophase detection: the continuous nature of development, unpredictable occlusion patterns, and the presence of natural confounders. While HCMS-Net substantially outperforms existing baselines, the error analysis indicates several directions for future improvement, including multi-modal sensing (e.g., depth or hyperspectral data) to resolve ambiguous color transitions, occlusion-aware data augmentation, and integration of temporal context from video sequences.

### 3.9 Cross-domain generalization performance validation

To rigorously evaluate the cross-domain generalization capability of HCMS-Net, we introduced a second, entirely independent self-built dataset of winter jujube. This species was deliberately chosen for its extreme visual dissimilarity from strawberry in morphology, texture, and cluster architecture, thereby constituting a stringent test for domain shift. The winter jujube dataset contains 13676 images acquired under natural field conditions, with challenges including dense occlusion, variable illumination, and small target sizes that mirror the strawberry scenario. Only the “ripe” and “unripe” class bounding box annotations were used, as fine-grained phenological stages are not defined for winter jujube.

Zero-shot transfer: The HCMS-Net trained exclusively on the strawberry dataset was directly evaluated on the winter jujube test set (1360 images) without any fine-tuning or domain adaptation. This tests the model’s ability to extract transferable visual features that generalize to an unseen species.

Cross-dataset fine-tuning: To assess the transfer efficiency of the learned representations, we fine-tuned the pre-trained model on progressively smaller subsets (100%, 50%, 10%) of the winter jujube training data and measured the recovery in mAP. We randomly sampled 10% (1250 images) and 50% (6155 images) of the winter jujube training set using a fixed random seed, while keeping the validation set (1360 images) intact. All fine-tuning runs were initialized from the strawberry pre-trained weights, trained for 50 epochs, and the model with the best mAP on the validation set was saved. Finally, the saved best model was evaluated on the held-out test set, and mAP@0.5 is reported.

The test set was not used in any training or hyperparameter selection process. All comparisons were conducted against YOLOv8, v10, v12, v13, and other state-of-the-art detectors under identical settings. The results are summarized in **Table 12**.

**Table 12:** Results of cross-domain generalization validation.

Model	Zero-shot mAP(%)	Fine-tune 10% data mAP(%)	Fine-tune 50% data mAP(%)	Fine-tune 100% data mAP(%)	Single image detection time		
					Pre (ms)	Inference (ms)	Post (ms)
YOLOv8n	5.4	58.6 ± 0.9	81.8 ± 0.7	93.3 ± 0.4	<0.1	0.2	0.6
YOLOv10n	7.9	52.6 ± 1.2	79.9 ± 0.8	92.8 ± 0.6	<0.1	0.1	0.5
YOLOv12n	5.1	54.9 ± 0.8	81.7 ± 0.6	92.8 ± 0.4	<0.1	0.1	0.8
YOLOv13n	4.2	57.4 ± 1.6	72.8 ± 0.9	92.4 ± 0.7	<0.1	0.3	0.5
Baseline(H-Net)	6.0	69.5 ± 0.7	85.8 ± 0.5	94.7 ± 0.4	<0.1	0.1	0.8
Ours(HCMS-Net)	3.8	77.7 ± 0.5	89.0 ± 0.3	96.2 ± 0.2	<0.1	0.1	0.7

All models exhibit extremely low zero-shot mAP due to the mismatch between the 5-class strawberry output and the 2-class jujube task, confirming the cross-dataset setup is genuinely challenging and free of label space overlap. Zero-shot performance (3.8%–7.9%) is uniformly poor, confirming that the strawberry and jujube domains are genuinely disjoint in both category space and visual appearance. Under 10% fine-tuning (1250 images), HCMS-Net achieves 77.7% mAP, which is 8.2 points higher than the best baseline (H-Net, 69.5%) and already surpasses the 50% fine-tuning results of several YOLO models. This validates that the MSCA and Conv2Former components learn highly transferable, domain-invariant features rather than strawberry-specific shortcuts. With full data (100%), HCMS-Net reaches 96.2%, consistently outperforming all baselines. Importantly, the strongest advantage appears in the extremely low-data regime, which is exactly the practical scenario where manual annotation for new crops is cost-prohibitive.

## 4 Conclusion

Identifying the phenological stages of crops is a key link in monitoring the growth status of crops, which serves as an indicator of crop growth status and also directly influences final crop yield. In this paper, we construct a real scene dataset covering five phenological stages for the problem of identifying each phenological stage of strawberries and SOD in unstructured environments. By combining HyperC2Net and YOLOv12n algorithm, hypergraph convolution and channel cascade were introduced to synthesize cross-level visual features, realize the semantic association from local to global, alleviate the limitation of insufficient small target features, and enrich the semantic depth of basic features. On this basis, the MSCA attention mechanism was introduced to aggregate local information and model the channel relationship, so as to realize spatial adaptive feature selection and enhancement. At the same time, without sacrificing accuracy, the lightweight detection head Conv2Former was integrated. By using large convolution kernels and deep convolutions, we broadened the receptive field while decreasing model complexity. Additionally, given the impact of bounding-box shape and scale on regression performance, we introduced the Shape-NWD loss function to improve the model’s sensitivity to minor localization errors and enhance its robustness.

All reported improvements are validated through 5-fold cross-validation with mean ± standard deviation, ensuring the model’s robustness and reliability for practical deployment. The final detection model achieved mAP, mAP@.5:.95 and F1 of 94.9%, 71.9% and 90% respectively, increasing by 4.1, 10.4 and 4 percentage points respectively. Among them, the AP values for the flowering period, juvenile fruit period, green fruit period, color change period and maturity period increased by 1%, 7.5%, 6.4%, 3.9% and 0.8%, respectively. Meanwhile, in terms of complexity, Model size, FLOPs and Parameters reached 6.4MB, 8.4G and 2.99M respectively, achieving a quite good balance in overall performance. The experimental results strongly proved the effectiveness of this method in accurately identifying the phenological stages of strawberries, as well as its sustainability and practical potential in long-term crop monitoring.

Despite these advances, this study still has several limitations: First, future research still needs to focus on improving the generalization ability under extreme conditions (such as strong radiation in daytime, weak radiation in fog, rainy weather, and other severe weather), so as to construct a phenological stage identification model covering the whole strawberry dataset to further optimize the early detection and adaptation ability in practical agricultural applications. Meanwhile, a complementary test set containing complete occlusion annotations (derived from multi-view consensus) can be created. These improvements will enhance the practicability of the research results and lay a solid foundation for the subsequent system platform deployment, thus promoting the development of remote observation and diagnosis technology under the background of precision agriculture. Secondly, the model needs to be further lightweight in order to be deployed to mobile devices to further promote the development of smart agriculture. Committed to a dedicated occlusion benchmark study as immediate future work, where we will annotate occlusion levels on a held-out test subset and report per-level performance across all models. Discussed potential strategies (e.g., occlusion-aware data augmentation, semi-supervised learning with unannotated occluded samples) for further improving heavy-occlusion performance. In addition, future research should explore more abundant multi-modal information such as depth image data, temporal features and spectral features on the basis of single modal data, and strive to use multi-modal data for more accurate strawberry phenology identification.

## Data Availability

The raw data supporting the conclusions of this article will be made available by the authors, without undue reservation.
